# Ra-223 SPECT for semi-quantitative analysis in comparison with Tc-99m HMDP SPECT: phantom study and initial clinical experience

**DOI:** 10.1186/s13550-017-0330-z

**Published:** 2017-10-03

**Authors:** Yoshiki Owaki, Tadaki Nakahara, Takeo Kosaka, Junichi Fukada, Atsuhiro Kumabe, Akira Ichimura, Mikoto Murakami, Kiyotaka Nakajima, Masahiro Fukushi, Kazumasa Inoue, Mototsugu Oya, Masahiro Jinzaki

**Affiliations:** 1 Department of Radiology, Keio University School of Medicine, 35 Shinanomachi, Shinjuku-ku, Tokyo, 160-8582 Japan; 2 Department of Urology, Keio University School of Medicine, 35 Shinanomachi, Shinjuku-ku, Tokyo, 160-8582 Japan; 3 Department of Radiological Sciences, Tokyo Metropolitan University, 7-2-10 Higashiogu, Arakawa-ku, Tokyo, 116-8551 Japan

**Keywords:** Ra-223, SPECT, Bone metastasis, Quantitation, Tc-99m

## Abstract

**Background:**

Image-based measurement of absorbed dose of Ra-223 dichloride may be useful in predicting therapeutic outcome in patients with castration-resistant prostate cancer (CRPC). In general, SPECT has been found to be more accurate than planar imaging in terms of lesion-based analysis. The aims of this study were to assess the feasibility and clinical usefulness of Ra-223 SPECT.

The energy spectrum of Ra-223 and SPECT images of a cylindrical phantom with a hot rod were obtained to determine the collimator candidates and energy window settings suitable for clinical Ra-223 SPECT (basic study A). Another phantom with a tube-shaped chamber and two spheres simulating bowel activity and metastatic lesions in the lumbar spine was scanned with medium-energy general-purpose (MEGP) and high-energy general-purpose (HEGP) collimators (basic study B). Ten patients with CRPC underwent SPECT imaging 2 h after Ra-223 injection successively with MEGP and HEGP collimators in random order for 30 min each. Lesion detectability and semi-quantitative analyses of bone metastasis (i.e. lesion-to-background ratio (LBR)) were performed compared to Tc-99m HMDP SPECT.

**Results:**

Basic study A revealed that an 84-keV photopeak ± 20% using the HEGP collimator offers better SPECT image quality than the other imaging conditions. Basic study B showed that uptake in one of the spheres was overestimated by overlapped activity of the tube-shaped chamber in planar imaging whereas the spheres had similar counts and significantly higher sphere-to-background ratio in SPECT. On both planar and SPECT images, HEGP gave higher image contrast than MEGP (*p* < 0.01). In the clinical study, Ra-223 SPECT at 84 keV ± 20% depicted more lesions with the HEGP than with the MEGP collimator (51 vs 36, *p* = 0.013). There was a positive correlation between LBR in Tc-99m SPECT and in Ra-223 SPECT (*r* = 0.67 with the MEGP and 0.69 with the HEGP collimator, *p* < 0.01). LBRs were significantly higher with the HEGP than with the MEGP collimator (*p* < 0.01).

**Conclusions:**

We recommended the use of the HEGP collimator at 84 keV ± 20% for Ra-223 SPECT imaging. Lesion-based semi-quantitative analysis in the human study revealed a good correlation between Ra-223 and Tc-99m HMDP SPECT in the early phase (2–3 h post injection).

**Electronic supplementary material:**

The online version of this article (10.1186/s13550-017-0330-z) contains supplementary material, which is available to authorized users.

## Background

Radium-223 is an alpha-emitting, bone-seeking radionuclide and is absorbed by bone tissue depending on bone remodelling [1–4]. Since bone metastasis of prostate cancer has high metabolic activity, it is considered that Ra-223 effectively accumulates in bone metastatic sites in patients with prostate cancer. In fact, Ra-223 dichloride is used for treatment of bone metastasis in patients with castration-resistant prostate cancer (CRPC) because bone metastasis causes skeletal-related events, such as bone pain and fractures, leading to deterioration of quality of life or death [5–7].

Although there has been solid evidence demonstrating the clinical utility of Ra-223 treatment, the survival curve of patients with CRPC receiving Ra-223 injection shows that not all benefited from this treatment [8]. In addition, the fact that Ra-223 therapy entails substantial costs and modest side effects, such as myelosuppression and digestive malfunction, indicates a potential need for selecting patients suitable for Ra-223 therapy. Given that its tumoricidal effect or side effects are likely to be dose-related, the image-based measurement of the absorbed dose delivered to the target would be useful for predicting therapeutic outcome or subsequent adverse events. Indeed, there have been some efforts to investigate suitable imaging techniques to depict Ra-223 uptake in metastatic sites [9–12].

Quantitative Ra-223 imaging is challenging because Ra-223, and its daughter nuclides have a wide range of energy spectrum, ranging from 80 to 500 keV [13–15], in addition to the limited number of emitted photons. The feasibility of quantitative planar imaging has been reported by some investigators [9–11], although lesion-based dosimetry is not an easy task on planar nuclear images because of the projection of several overlying structures, such as the intestine, in a planar image. The area depicting the lumbar spine may therefore contain some portion of physiologic bowel uptake; prescribing information provided by the U.S. Food and Drug Administration (FDA) shows that because of excretion of Ra-223 into the small intestine, radioactivity in the intestine is as high as radioactivity in the bone at 4 h post-injection (approximately 61 vs 49%) [16]. Taken together with the fact that bone metastasis in prostate cancer tends to occur in the pelvis and lumbar spine, Ra-223 SPECT may offer advantages for treatment assessment of individual lesion. However, to our knowledge, there has been no report regarding the technical feasibility of Ra-223 SPECT imaging.

In Ra-223 imaging, technical elaboration is more important in SPECT than in planar scintigraphy due to the limited number of signals to visualise bone accumulation. Among technical factors, the energy window setting is known to affect the estimation of Ra-223 uptake; according to a Monte Carlo simulation study, it is optimal to set the energy peak at 84 keV using a medium-energy general-purpose (MEGP) collimator [9]. Hindorf et al. investigated 82, 154 and 270 keV energy peaks, but they only recommended the use of 82 keV [12]. However, the use of a high-energy general-purpose (HEGP) collimator has never been considered. We previously reported the advantage of the HEGP collimator for Sr-89 imaging [17]. We found that characteristic X-rays of lead, which are produced by interaction between high-energy photons and lead in the collimator, have positive impact on the image quality in Sr-89 imaging. In addition, since photon penetration is reduced when using thick collimators, the HEGP collimator is expected to decrease more background counts than the MEGP collimator in Ra-223 imaging.

In this study, we determined an optimal energy window setting and collimator choice for Ra-223 SPECT, based on the energy spectrum of Ra-223 and a phantom study. In addition, human Ra-223 SPECT was semi-quantitatively compared to Tc-99m hydroxymethylene diphosphonate (HMDP) SPECT on a per-lesion basis. Thus, the aims of this study were to assess the feasibility and clinical usefulness of Ra-223 SPECT.

## Methods

### Acquisitions of the energy spectrum and a cylindrical phantom image to determine energy window settings and collimator choice for Ra-223 SPECT imaging (basic study A)

**Fig. 1:**
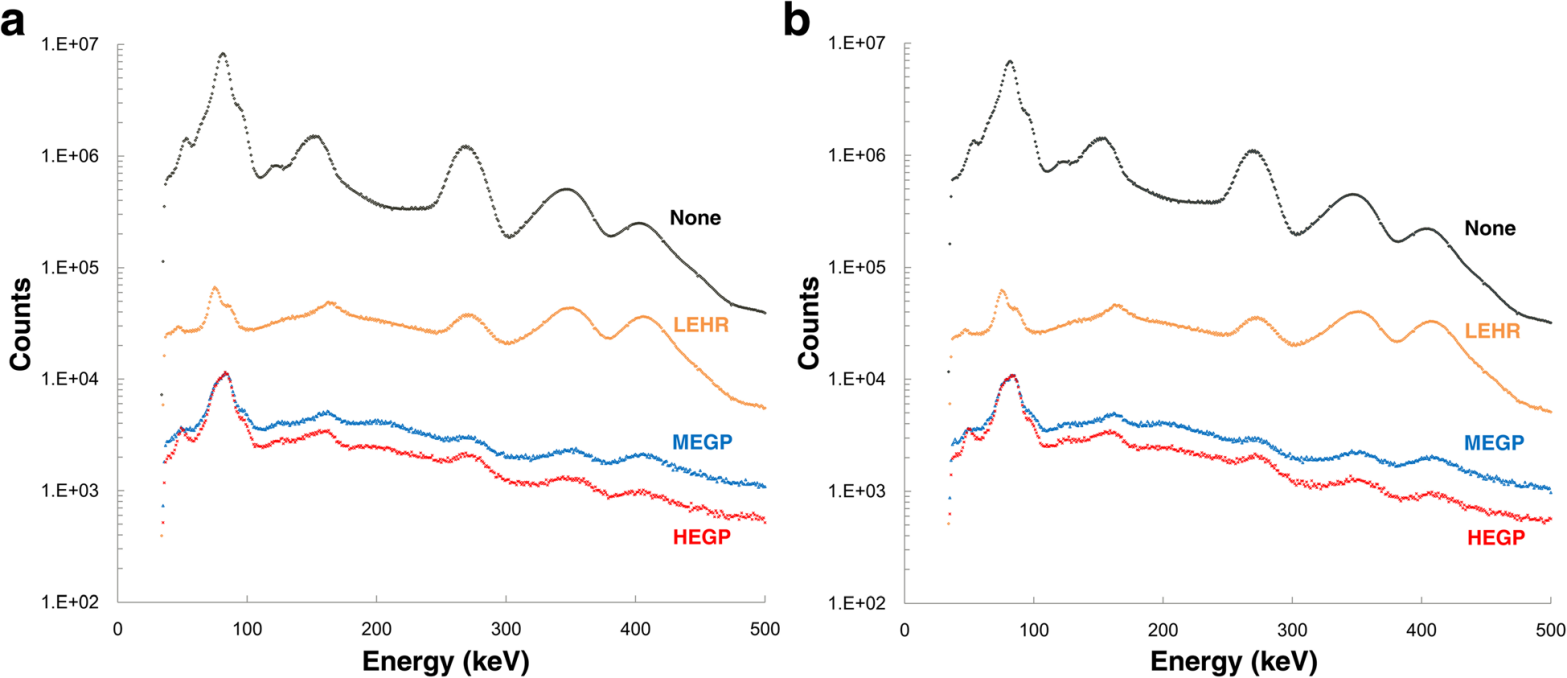
Energy spectra of a Ra-223 vial around the air (**a**) and a Ra-223 vial in the chamber filled with iodine contrast media (CT value, 350 HU) (**b**) with the LEHR, MEGP and HEGP collimators or without any of collimator. *LEHR* low-energy high-resolution, *MEGP* medium-energy general purpose, *HEGP* high-energy general purpose

The energy spectra of Ra-223 with low-energy high-resolution (LEHR), MEGP or HEGP collimators and without any collimator were obtained using a dual-headed SPECT system (Discovery NM630, GE Healthcare, Milwaukee, WI). The thickness of the detector crystal was 3/8 in. Hole diameter, hole length and lead thickness of the collimator were 3.0, 58 and 1.1 mm for MEGP and 4.0, 66 and 1.8 mm for HEGP, respectively. Each of the two vials containing Ra-223 solution (1.1 MBq, 2.0 mL) was carefully placed at a distance of 100 mm from the detector under the different conditions where the vials were set around the air or in the chamber filled with iodine contrast media (CT value, 350 HU), and each energy spectrum was then obtained (Fig. 1). The energy spectrum < 500 keV was obtained for 30 min.

Image quality was assessed using a cylindrical phantom filled with water (diameter, 200 mm; height, 210 mm) in which a cylindrical rod (diameter, 45 mm; height, 200 mm) filled with 2.0 kBq/mL of Ra-223 was embedded along the phantom axis (Additional file 1: Figure S1). The radio concentration of the rod was determined based on the phantom study by Hindorf et al. [12]. SPECT/CT scans were performed with MEGP and HEGP collimators using the same protocol (matrix, 64 × 64; pixel size, 8.8 mm; scan orbit, body contour; frames per detector, 30 (6° steps); acquisition time per frame, 60 s; total acquisition time, 30 min). Total acquisition time was determined based on the balance of count statistics and patient durability in our clinical study described later.

First, the energy window setting around 84 keV was evaluated. We focused on image noise and contrast in image quality of Ra-223 SPECT because of the limited injected dose. As studied in Sr-89 imaging [17], we evaluated the impact of characteristic X-rays of lead (75 keV (Kα) and 85 keV (Kβ) [18]) on image quality of Ra-223 SPECT by comparing the energy window widths between ± 20% and ± 10%. The latter width has been used so far [9, 12], although these characteristic X-rays have not been fully included.

The SPECT data with the two energy window widths were reconstructed using 3-dimensional ordered subset expectation maximisation (3D OSEM) algorithm (five subsets, 10 iterations). Butterworth filter was used for image noise reduction (cutoff frequency, 0.20 cycle/cm; order, 10). Attenuation or scatter correction was not performed. Circular regions-of-interest (ROIs) (diameter, 45 mm) were carefully placed on the hot rod in a transaxial slice (slice thickness, 8.8 mm) with a reference of the corresponding CT (Additional file 1: Figure S1). Background counts (BKG) were also measured using the same 8 ROIs, avoiding high activity around the hot rod. Then, the quality of SPECT images was assessed with the following parameters:

- Hr = ROI counts in the hot rod
- BKG = averaged ROI counts in the background
- SD = standard deviation of BKG
- Hot rod-to-background ratio (HBR) = Hr/BKG
- Contrast-to-noise ratio (CNR) = (Hr–BKG)/SD

Second, the energy window regarding the other peaks was set at 154 keV ± 10% and 269 keV ± 5% according to previous reports [9, 12]. Similarly, Hr, BKG, HBR and CNR were measured. The procedure of the parameter measurement was repeated 10 times (i.e. 10 arbitrary transaxial slices) obtained with the two collimators.

### SPECT acquisition of a modified body phantom simulating bowel activity and lumbar spine metastases compared to planar imaging for clinical Ra-223 SPECT (basic study B)

**Fig. 2:**
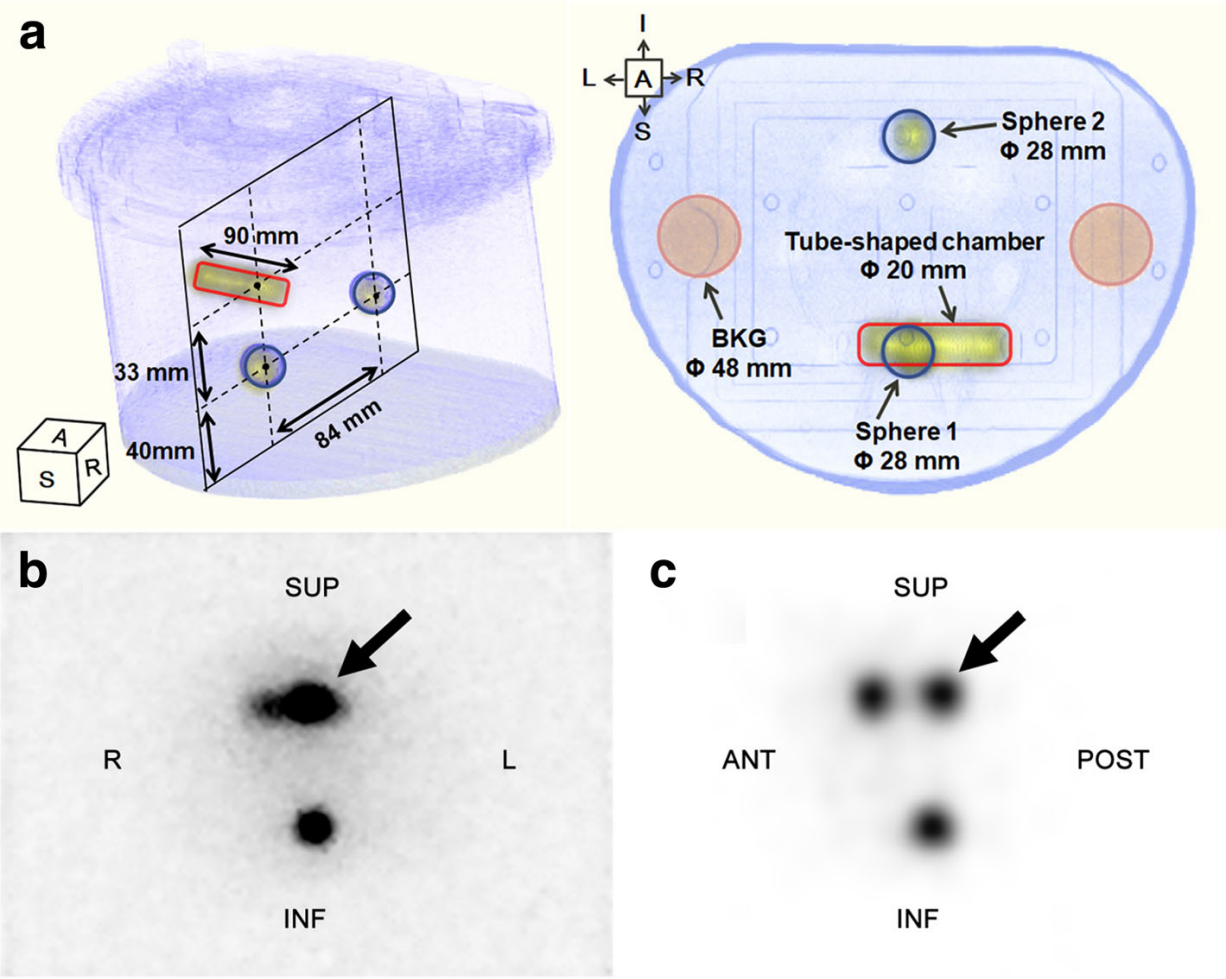
Configuration of a modified body phantom simulating bowel activity and lumbar spine metastases (**a**). Uptake in sphere 1 (thick arrows) is overlaid with uptake of the tube-shaped chamber in the anteroposterior planar image (**b**) but not in the mid-sagittal plane of SPECT (**c**). Sphere 2 is used as reference. *ANT* anterior, *SUP* superior, *POST* posterior, *INF* inferior, *R* right, *L* left, *BKG* background

A modified body phantom simulating bowel activity and lumbar spine metastases was developed and scanned. The configuration of the phantom is shown in Fig. 2a. The phantom was filled with water, and two identical 28 mm spheres (11.5 cm^3^) with 15.0 kBq/mL of Ra-223 and attenuation medium (350 HU) and a tube-shaped chamber with 15.0 kBq/mL of Ra-223 were embedded. The spheres were set in the same longitudinal axis. One of them (sphere 1) was set in the same anteroposterior axis as a tube-shaped chamber so that uptake of the sphere is overlaid by uptake of the chamber on the planar image. The other sphere (sphere 2) was used as reference. Planar static data were obtained for 30 min in a 128 × 128 matrix. Image acquisition and reconstruction for SPECT were the same as those for a cylindrical phantom.

In this phantom study, the following parameters were evaluated; first, using the data of sphere 2, the threshold for spherical volume of interest (VOI) of bone metastasis for clinical Ra-223 SPECT was determined by comparing the sphere volumes on CT and SPECT. Second, the uptake ratio of sphere 1 to sphere 2 was assessed using a 28-mm circular ROI in the anteroposterior view of the planar image and using the threshold method in SPECT. Third, the sphere-to-background ratio for sphere 2 (SBR) was compared between the planar and SPECT images using 48-mm circular background ROIs for the planar image and 48-mm spherical background VOIs for the SPECT (Fig. 2a). Finally, the phantom was repeatedly scanned to evaluate the linearity between radio-concentration and SPECT count in sphere 2.

### Direct comparison of clinical Ra-223 and Tc-99m HMDP SPECT images

Both Ra-223 treatment and image acquisition of Ra-223 in patients with prostate cancer were approved by the institutional review board in Keio University Hospital (approval number 20160203). The study was conducted in accordance with the Declaration of Helsinki. The study protocol was registered at the University Hospital Medical Information Network (UMIN000024274). Written informed consent was obtained from 10 patients with prostate cancer who had a bone metastasis diagnosis confirmed with Tc-99m HMDP SPECT/CT.

Injection dose of Ra-223 dichloride solution was 55 kBq/kg per cycle. Image acquisition was performed after the first injection during six cycles of Ra-223 treatment in all patients. One-bed Ra-223 SPECT scanning was performed in an area where the greatest bone metabolism was revealed in Tc-99m HMDP SPECT. SPECT data were collected 2 h after Ra-223 injection successively with the MEGP and HEGP collimators for 30 min each. Since the difference in uptake time from injection to image acquisition could affect image contrast between the two successive SPECT acquisitions (120 vs 150 min), image acquisition was performed first with the MEGP collimator for five patients and with the HEGP collimator for the remaining five. The energy windows were set at 84 keV ± 20% according to the results of the phantom study. Acquisition and reconstruction parameters were identical to those used for the phantom images.

Tc-99m HMDP SPECT/CT was performed 3 h after injection using the LEHR collimator at 141 keV ± 10%. Tc-99m HMDP SPECT data were also reconstructed on a 128 × 128 matrix (pixel size, 4.4 mm) with 3D OSEM (five subsets; 10 iterations) and Butterworth filter (cutoff frequency, 0.33 cycle/cm; order, 10) using attenuation and scatter correction (dual-energy window method) and resolution recovery with Evolution for Bone (GE Healthcare).

With a reference of CT images, the LBR was calculated for metastatic lesions showing increased Ra-223 uptake at least two times greater than uptake in the normal spine with both the MEGP (LBRme) and HEGP (LBRhe) collimators. The LBR for Tc-99m HMDP SPECT was also calculated (LBRtc). VOIs were placed on the metastatic lesions to encompass the selected metastasis at a contour level of 60% of the highest count. For background VOIs, spherical VOIs (50 cm^3^) were carefully drawn within the liver parenchyma with reference to the corresponding CT images, avoiding the large hepatic vein. Then, the LBR was measured as mean counts in the lesion ROI divided by mean counts in the background ROI.

### Statistical analysis

All statistical analyses were performed using JMP 12.0.1 software (SAS Institute Inc., Cary, NC). All data were expressed as median and range (minimum to maximum) and analysed with non-parametric methods. Comparisons of Hr, BKG, HBR and CNR for the cylindrical phantom between the energy window of 84 keV ± 20% and ± 10% were performed using the Wilcoxon signed-rank test. After selecting the optimal energy window, around 84 keV, comparisons of Hr, BKG, HBR and CNR among the three energy windows were performed using Friedman’s test, and when significant differences were found, the Steel-Dwass test was performed on each pair. Comparisons of SBR for the modified body phantom between planar vs SPECT or MEGP vs HEGP were also performed using the Wilcoxon signed-rank test.

Regarding the clinical study, detection of bone metastasis was compared between Ra-223 SPECT and Tc-99m HMDP SPECT using the Wilcoxon signed-rank test. The relationships between LBRme or LBRhe vs LBRtc were assessed using linear regression analysis. The comparison between LBRme and LBRhe was performed using paired *t* test. The differences at the 95% confidence level (*p* < 0.05) were considered to be statistically significant.

## Results

### Acquisitions of the energy spectrum and a cylindrical phantom image to determine energy window settings and collimator choice for Ra-223 SPECT imaging (basic study A)

The energy spectra of Ra-223 with the MEGP or HEGP collimators and without any collimator are shown in Fig. 1. The peak at 84 keV when using HEGP has a wide width probably because of the characteristic lead X-rays occurring by interaction of primary photons and lead. Detector sensitivity under the use of the HEGP collimator relative to the use of the MEGP collimator and the photopeak height at 154, 269, 351 and 402 keV relative to that at 84 keV under the use of the HEGP collimator are shown in Table 1. The existence of some attenuation medium surrounding the vial had limited impact on the energy spectrum, suggesting that patient body is unlikely to affect the energy spectrum.

**Table 1:** Energy spectra of a Ra-223 vial with MEGP and HEGP

Energy peak	Relative detector sensitivity with HEGP to MEGP collimator		Relative peak height to that at 84 keV with HEGP		% decrease in sensitivity by attenuation medium	
	Attenuation medium (−)	Attenuation medium (+)^a^	Attenuation medium (−)	Attenuation medium (+)^a^	MEGP	HEGP
84	99.5	98.3	NA	NA	2.9	2.3
154	70.2	74.1	29.3	31.2	3.4	1.1
269	69.8	69.9	18.9	18.8	4.3	4.1
351	52.0	56.8	11.2	12.0	5.5	0.9
402	47.5	46.4	8.8	8.8	2.4	4.1

^a^CT value of a chamber containing attenuation medium enclosing the vial was 350 HU

*NA* not applicable, *MEGP* medium-energy general purpose, *HEGP* high-energy general purpose

**Fig. 3:**
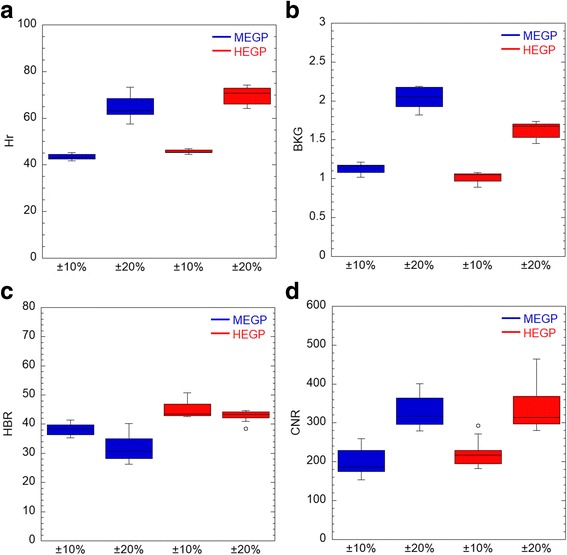
**a**–**d** Comparison of SPECT image quality of the cylindrical phantom between the energy window settings of 84 keV ± 20% and 84 keV ± 10%. SPECT counts in hot rod (Hr) and background (BKG), and the standard deviation of BKG (SD) were measured. CNR and HBR are defined in the text. *MEGP* medium-energy general purpose, *HEGP* high-energy general purpose, *CNR* contrast-to-noise ratio, *HBR* hot rod-to-background ratio

Figure 3 shows the comparison of SPECT image quality of the cylindrical phantom between the energy window settings of 84 keV ± 20% and 84 keV ± 10%. Hr, BKG and CNR were significantly higher at 84 keV ± 20% than at 84 keV ± 10%, irrespective of collimators. It should be noted that HBR was similar between the two window settings whereas CNR at 84 keV ± 20% was approximately 50% higher than CNR at 84 keV ± 10% (*p* < 0.01). Based on these results, the optimal window width around 84 keV for Ra-223 SPECT was determined to be 84 keV ± 20%, and then, further analyses were performed using this window setting.

Additional file 1: Figure S2 shows Hr, BKG, HBR and CNR at 84 keV ± 20%, 154 keV ± 10% and 269 keV ± 5%. Hr at 84 keV was significantly higher with the HEGP than with the MEGP collimator (*p* < 0.05) whereas Hrs at 154 keV and 269 keV were significantly lower with the HEGP than with the MEGP collimator (*p* < 0.05). BKG was significantly lower with the HEGP collimator than with the MEGP collimator at all the energy windows (*p* < 0.01), as visually shown in Additional file 1: Figure S1B and C. HBR at 84 keV was significantly higher with the HEGP than with the MEGP collimator (*p* < 0.01). Based on these results, further analyses were performed at 84 keV ± 20%.

### SPECT acquisition of a modified body phantom simulating bowel activity and lumbar spine metastases compared to planar imaging for clinical Ra-223 SPECT (basic study B)

On SPECT images of a modified body phantom, the volumes of sphere 2 (11.5 cm^3^) with thresholding at 50, 60, 70 and 80% of the maximum sphere value were 15.8, 9.0, 6.2 and 1.4 cm^3^ when using the MEGP collimator and 17.9, 12.4, 11.0 and 9.0 cm^3^ when using the HEGP collimator, respectively. In order to match VOI size with true lesion size, thresholding at 60% of the maximum was used for further phantom and clinical SPECT studies.

**Fig. 4:**
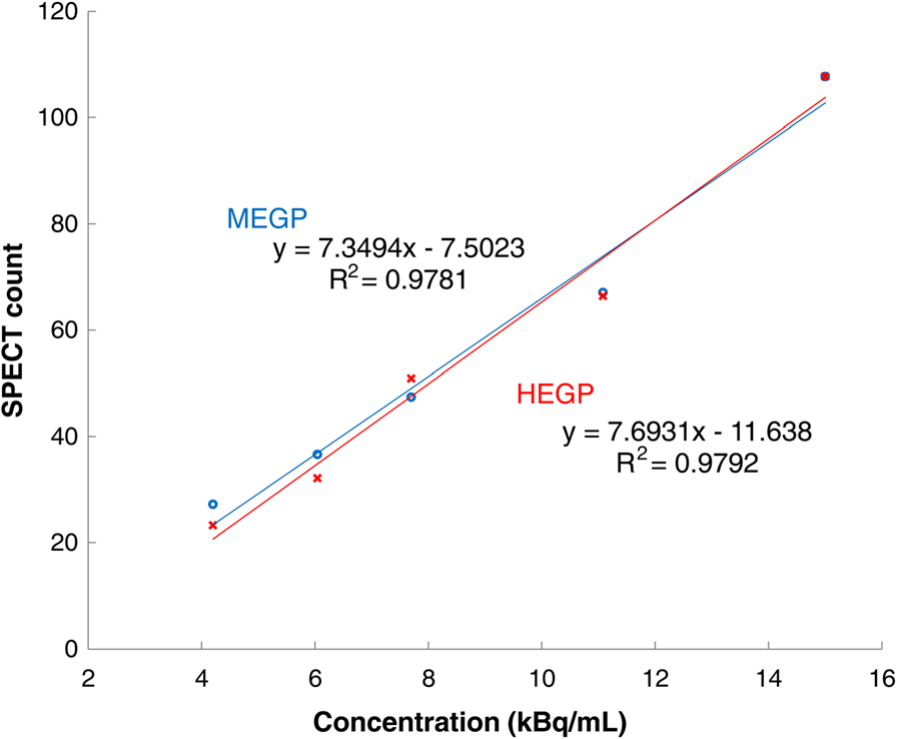
Relationship between radio-concentration of Ra-223 and SPECT counts at 84 keV ± 20% when using the MEGP or HEGP collimators. *MEGP* medium-energy general purpose, *HEGP* high-energy general purpose

Figure 2b, c shows a planar image of the phantom in the anteroposterior view and the mid-sagittal plane of the SPECT image in which the spheres are most clearly shown, respectively. Irrespective of the collimator used, the uptake value of sphere 1 was overestimated by approximately 45% on the planar image whereas it was almost identical to the uptake value of sphere 2 on the SPECT image (Table 2). On the other hand, SBR in SPECT was more than twice as high as in planar imaging (*p* < 0.001). In both planar and SPECT imaging, SBR was also significantly higher with HEGP than with MEGP (*p* < 0.01). Figure 4 shows a good linearity between the radio-concentration of Ra-223 and SPECT counts at 84 keV ± 20% with both the MEGP and HEGP collimators.

**Table 2:** Uptake ratio of sphere 1 to sphere 2 and sphere-to-background ratio (SBR) in a modified body phantom

Ratio	Acquisition	Collimator		*P*
		MEGP	HEGP	
Sphere 1/sphere 2				
	Planar	1.45	1.47	NA
	SPECT	1.05	0.99	NA
SBR (sphere 2)				
	Planar*	13.3 (12.3–14.2)	15.6 (14.1–17.0)	0.0019
	SPECT*	34.5 (30.4–35.3)	39.2 (35.9–43.1)	0.0058

Configuration of the phantom and location of the spheres are described in Fig. 1

Methods of measuring background and sphere counts are described in text

*P < 0.001 for comparison between planar and SPECT imaging

*NA* not applicable, *MEGP* medium-energy general-purpose, *HEGP* high-energy general-purpose

### Direct comparison of clinical Ra-223 and Tc-99m HMDP SPECT images

**Fig. 5:**
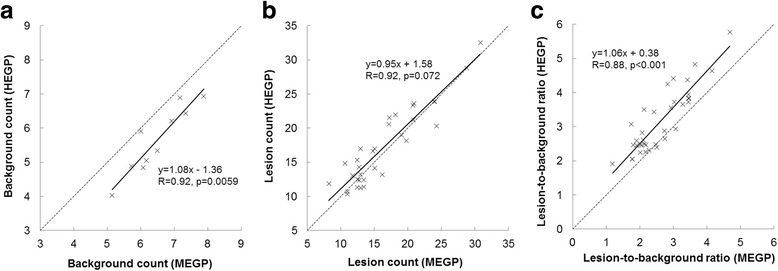
**a**–**c** Comparison of background count (patient-based), lesion count and lesion-to-background ratio between the MEGP and HEGP collimators. *MEGP* medium-energy general purpose, *HEGP* high-energy general purpose

**Fig. 6:**
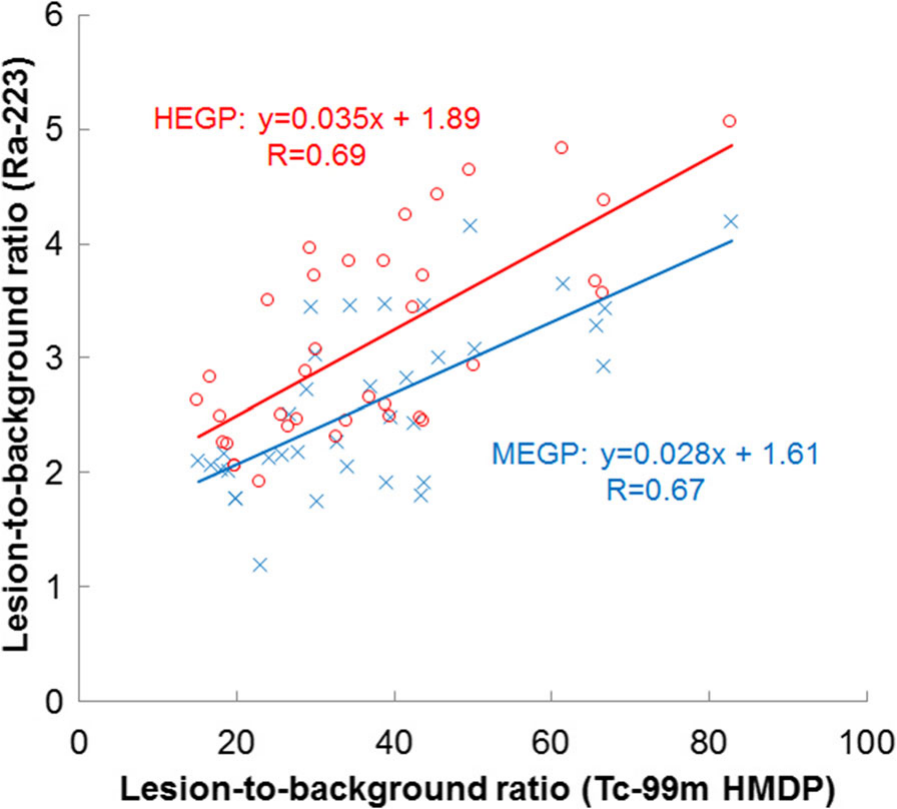
Relationship of lesion-to-background ratio between Tc-99m HMDP SPECT and Ra-223 SPECT with the MEGP or HEGP collimators. *MEGP* medium-energy general purpose, *HEGP* high-energy general purpose, *HMDP* hydroxymethylene diphosphonate

**Fig. 7:**
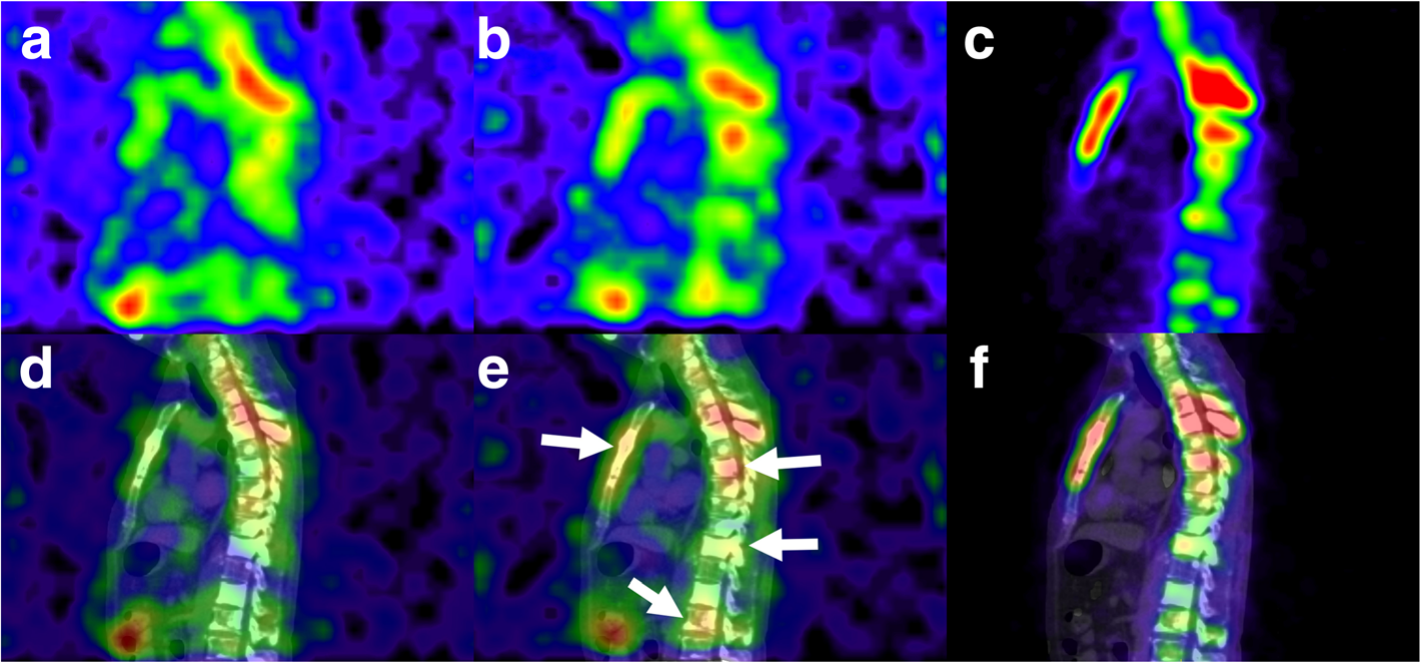
Comparison of Ra-223 SPECT with the MEGP (**a**) or HEGP collimators (**b**) and Tc-99m HMDP SPECT (**c**) in a patient with prostate cancer with bone metastases (patient no. 3). The corresponding SPECT/CT fused images are shown in **d**, **e**, and **f**. It should be noted that Ra-223 SPECT depicted more lesions with the HEGP than with the MEGP collimator (arrows), even though the SPECT scan with HEGP was performed prior to the scan with MEGP. *HMDP* hydroxymethylene diphosphonate, *MEGP* medium-energy general purpose, *HEGP* high-energy general purpose, *HMDP* hydroxymethylene diphosphonate

**Fig. 8:**
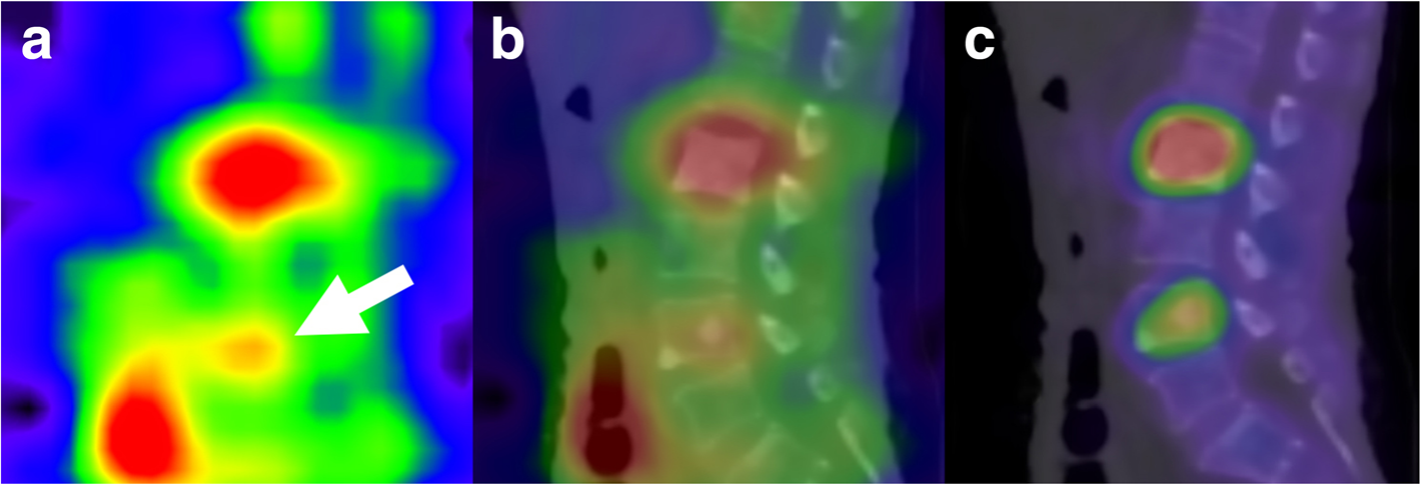
Coexistence of bowel uptake and bone metastasis in the lumbar spine revealed with Ra-223 SPECT (patient no. 6). SPECT (**a**) and SPECT/CT fused images (**b**) show that uptake in a bone metastasis at the fourth lumbar vertebra (arrow) is weak compared to adjacent bowel uptake. The corresponding Tc-99m HMDP SPECT/CT image (**c**) shows no bowel uptake. *HMDP* hydroxymethylene diphosphonate

In the evaluated 10 patients, Tc-99m HMDP SPECT depicted more lesions than Ra-223 SPECT with each collimator (84 vs 51 for HEGP, *p* < 0.01; 84 vs 36 for MEGP, *p* < 0.01). In addition, Ra-223 SPECT depicted more lesions with the HEGP than with the MEGP collimator (51 vs 36, *p* = 0.013), and no lesion was only detected with the MEGP collimator. Thus, semi-quantitative analysis was performed for the 36 metastatic lesions to directly compare the MEGP and HEGP collimators (Table 3). The comparison of background count (patient-based), lesion count, and LBR (lesion-based) between these collimators is shown in Fig. 5. The lesion count was similar between the HEGP and MEGP collimators whereas the background count was significantly lower with the HEGP than with the MEGP collimator (*p* < 0.01). The relationships between LBRme or LBRhe and LBRtc are shown in Fig. 6. There were positive correlations between LBRtc and LBRme (*r* = 0.67, *p* < 0.01) or LBRhe (*r* = 0.69, *p* < 0.01). LBRhe was significantly higher than LBRme (*p* < 0.01). An example of Ra-223 SPECT with the MEGP and HEGP collimators compared to Tc-99m HMDP SPECT in a patient with bone metastasis is presented in Fig. 7. Bowel uptake was seen in nine patients; six of which showed greater bowel activity than uptake in metastatic sites (Table 3). A representative case in which bowel uptake was successfully separated from disease uptake using SPECT is shown in Fig. 8.

**Table 3:** Direct comparison of clinical Ra-223 and Tc-99m HMDP SPECT images

Patient no.	Age (year)	Weight (kg)	Injected activity (MBq)	Scan area	Bowel activity in scan area	Lesion site for LBR measurement	Order of collimator used for Ra-223 SPECT	Number of visible lesion sites		
								Tc	MEGP	HEGP
1	73	61	3.44	Chest	–	T12	MEGP, HEGP	5	1	3
2	68	64	3.35	Chest	+	T3, T8, L2, right 4th rib, left third rib	MEGP, HEGP	10	5	7
3	69	59	3.25	Chest	++	T3, T6, T10, L1, sternum	HEGP, MEGP	14	5	9
4	73	71	3.62	Pelvis	+++	L4, Iliac bone	HEGP, MEGP	5	2	4
5	67	61	3.17	Chest	+++	T3, T6, T8, T11, L2, L3, right 7th rib, left 6th rib, right clavicle	MEGP, HEGP	19	9	11
6	78	41	2.13	Abdomen	+++	T11, L2, L4, right 7th rib	MEGP, HEGP	9	4	5
7	69	67	3.68	Pelvis	+++	Sacrum	HEGP, MEGP	2	1	1
8	76	54	3.07	Abdomen	+++	T8, L2, L4	HEGP, MEGP	5	3	4
9	83	61	3.22	Abdomen	+++	T10, L4	MEGP, HEGP	7	2	2
10	74	68	3.70	Abdomen	++	T4, T9, L2, left 2nd rib	HEGP, MEGP	8	4	5

Bowel activity was classified as (−) no uptake, (+) weaker than uptake in the metastatic sites, (++) comparable to uptake in the metastatic sites, (+++) stronger than uptake in the metastatic sites

*T* thoracic spine, *L* lumbar spine, *MEGP* medium-energy general-purpose, *HEGP* high-energy general-purpose, *LBR* lesion-to-background ratio

## Discussion

Ra-223 dichloride has been used for targeted cancer therapy in patients with prostate cancer with bone metastasis because of the potential of alpha particles for breaking double-strand DNA in metastatic tumour cells. Since Ra-223 is a bone-seeking radionuclide, the delivered radiation dose to the bone metastasis in Ra-223 therapy is partly dependent on local bone metabolism. Although it is known that bone metastasis of prostate cancer primarily occurs in the pelvis and lumbar spine, the evaluation of quantitative uptake in these areas would be difficult because of bowel uptake on planar images. It was reported that Tc-99m MDP uptake was correlated with Ra-223 dichloride uptake on planar images in nine patients with prostate cancer with 24 bone metastases, including a small number of lesions in the pelvis (*n* = 5) or the lumbar spine (*n* = 2) [10]. In order to separate pathological bone uptake from bowel uptake, there is a need for SPECT imaging as seen in Figs. 2 and 8. In our study, 90% (9/10) of the patients showed bowel uptake in the scan areas (Table 3). To the best of our knowledge, the present study was the first to demonstrate the feasibility of Ra-223 SPECT imaging to allow for semi-quantitative analysis, compared to Tc-99m HMDP SPECT imaging.

There are several advantages of HEGP over MEGP for Ra-223 imaging. First, according to the energy spectra using the LEHR, MEGP, and HEGP collimators, HEGP had the lowest detection count, followed by MEGP and then LEHR (Fig. 1). The results of a cylindrical phantom revealed that the spectral differences were due to the differences of the shielding effect between these collimators (Fig. 3). Second, the counts of the vial or hot rod around 84 keV were similar or even greater with HEGP despite the shielding effect. This is because the greatest peak of the Ra-223 energy spectrum is coincidentally close to the peaks of these characteristic X-rays. The use of a thicker collimator would contribute to the efficient yield of characteristic X-rays. For these reasons, HEGP would give a higher image contrast than MEGP. Indeed, the clinical study clearly showed a higher detection rate with HEGP than with MEGP.

Regarding the energy window width around 84 keV for Ra-223 imaging, we used 40% instead of 20% that has been used in previous studies [9, 12]. Our reasoning is as follows. Hindorf et al. recommended 30 min of acquisition for planar static images for an injected activity of 100 kBq/kg [12]. Together with the injected dose in our study, SPECT imaging for 30 min may have been insufficient to obtain count statistics for acceptable image quality. However, image acquisitions over 60 min could not be tolerated by patients with bone metastases. Therefore, we determined the acquisition time for SPECT with MEGP and HEGP collimators to be 30 min each. In this situation, due to limited count statistics, background noise greatly affects image quality especially in SPECT. Inclusion of lead characteristic X-rays was therefore considered to increase image acquisition counts without significantly decreasing the image contrast between lesion and background, as shown in Fig. 3.

Neither attenuation nor scatter corrections were performed for Ra-223 SPECT in the present study. In clinical practice, available scatter correction methods include energy-dependent scatter corrections (e.g. dual- or triple-energy window methods). In such corrections, the sub-window setting (centre and width of energy window) should be determined based on the spectral waveform. However, considering the bottom of the spectrum of Ra-223 over the whole energy level (Fig. 1), there is a substantial amount of background counts, comprising not only scatter but penetrated photons, compared to counts from primary photons. This is quite different from Tl-201, Tc-99m and I-123 imaging in which energy-dependent scatter correction is often effective. Therefore, it is unknown whether energy-dependent scatter correction is suitable for Ra-223 imaging based on our basic studies. In addition, sensitivity with Ra-223 is poor due to limited injected radioactivity (at most 10 MBq), leading to the low count statistics. Therefore, count reduction by scatter correction significantly affects image quality in Ra-223 imaging. Moreover, attenuation was not performed because attenuation correction using a linear attenuation coefficient of radionuclides other than for clinically relevant radionuclides is challenging. In addition, attenuation without scatter correction may induce overcorrection.

We examined the correlation between HMDP and Ra-223 uptake on a per-lesion basis. This finding was supported by the finding of the previous study that Tc-99m MDP percent uptake was correlated with Ra-223 dichloride percent uptake on the planar image [10]. However, our study showed that Tc-99m HMDP SPECT was more sensitive, and LBRme or LBRhe were about 10-fold lower than LBRtc, which is probably due to the difference in the imaging condition under which Tc-99m SPECT was obtained using attenuation and scatter correction and resolution recovery, whereas no corrections were used for Ra-223 SPECT. High background in Ra-223 SPECT due to substantial amount of scattered and penetrated photons accounted for very low LBR compared to Tc-99m SPECT. The difference in spatial resolution would also contribute to these results. Although further improvement of imaging technique is therefore necessary to assess whether biokinetics in metastatic bone tissue is similar between Radium and HMDP, the correlation of SPECT-derived uptake values we found in this study would encourage us to perform further studies on quantitative Ra-223 SPECT.

This study has several limitations. First, we could not obtain a planar image in this clinical study because of the patients’ inability to tolerate acquisition times over 60 min, as previously discussed. Therefore, we did not perform a direct comparison between planar and SPECT imaging and instead compared the performance of MEGP and HEGP in SPECT. Second, Ra-223 SPECT was only evaluated in the early phase because it was not feasible for all eligible patients to return to our hospital only for SPECT scanning. Further studies are needed to improve the detection and dose evaluation of bone metastases using Ra-223 SPECT.

## Conclusions

We recommended the use of the HEGP collimator at 84 keV ± 20% for Ra-223 SPECT imaging based on the energy spectrum of Ra-223 and a phantom study. The lesion-based, semi-quantitative analysis in the human study revealed a good correlation between Ra-223 and Tc-99m HMDP SPECT in the early phase (2–3 h post injection). Ra-223 SPECT is technically feasible and clinically useful for lesion-based analysis even in the pelvis and lumbar spine. Given that the tumoricidal effect of Ra-223 is likely to be dose-related, further studies on the assessment of the absorbed dose delivered to individual metastatic lesions using Ra-223 SPECT may lead to better prediction of therapeutic outcome and contribute to patient selection.

## Additional file

Additional file 1:
**Figure S1.** Configuration of a cylindrical phantom (A) and summed transaxial images (slice thickness, 14 cm) at 84 keV with MEGP (B) and HEGP (C) collimators. The phantom was filled with water, and a hot rod (2.0 kBq/mL) was embedded along the phantom axis. Circular regions-of-interest were placed on the hot rod and background. MEGP, medium-energy general purpose; HEGP, high-energy general purpose. **Figure S2.** SPECT counts in a hot rod (Hr) and background (BKG), hot rod-to-background ratio (HBR) and contrast-to-noise ratio (CNR) at 84 keV ± 20%, 154 keV ± 10% and 269 keV ± 5%. (DOCX 2051 kb)

## Abbreviations

3D OSEM: 3-Dimensional ordered subset expectation maximisation
BKG: Background counts
CNR: Contrast-to-noise ratio
CRPC: Castration-resistant prostate cancer
HBR: Hot rod-to-background ratio
HEGP: High-energy general purpose
HMDP: Hydroxymethylene diphosphonate
Hr: ROI counts in the hot rod
LBR: Lesion-to-background ratio
LEHR: Low-energy high-resolution
MDP: Methylene diphosphonate
MEGP: Medium-energy general purpose
ROI: Regions-of-interest
SPECT: Single-photon emission computed tomography
SPECT/CT: Single-photon emission computed tomography/computed tomography
SRE: Skeletal-related events
