# Supplementary material for: Ra-223 SPECT for semi-quantitative analysis in comparison with Tc-99m HMDP SPECT: phantom study and initial clinical experience
Source: EJNMMI Res. 2017 Oct 3;7:81. doi: 10.1186/s13550-017-0330-z (PMC5626671; doi:10.1186/s13550-017-0330-z)
Supplement: Additional file 1: — Figure S1. Configuration of a cylindrical phantom (A) and summed transaxial images (slice thickness, 14 cm) at 84 keV with MEGP (B) and HEGP (C) collimators. The phantom was filled with water, and a hot rod (2.0 kBq/mL) was embedded along the phantom axis. Circular regions-of-interest were placed on the hot rod and background. MEGP, medium-energy general purpose; HEGP, high-energy general purpose. Figure S2. SPECT counts in a hot rod (Hr) and background (BKG), hot rod-to-background ratio (HBR) and contrast-to-noise ratio (CNR) at 84 keV ± 20%, 154 keV ± 10% and 269 keV ± 5%. (DOCX 2051 kb) [file 13550_2017_330_MOESM1_ESM.docx]

**Supplement**

**Title:** Ra-223 SPECT for semi-quantitative analysis in comparison with Tc-99m HMDP SPECT: phantom study and initial clinical experience

**Authors:** Yoshiki Owaki^1,3^, Tadaki Nakahara^1^, Takeo Kosaka^2^, Junichi Fukada^1^, Atsuhiro Kumabe^1^, Akira Ichimura^1^, Mikoto Murakami^1^, Kiyotaka Nakajima^1^, Masahiro Fukushi^3^, Kazumasa Inoue^3^, Mototsugu Oya^2^, Masahiro Jinzaki^1^

^1^Department of Radiology, Keio University School of Medicine, 35 Shinanomachi, Shinjuku-ku, Tokyo, 160-8582, Japan.

^2^Department of Urology, Keio University School of Medicine, 35 Shinanomachi, Shinjuku-ku, Tokyo, 160-8582, Japan.

^3^Department of Radiological Sciences, Tokyo Metropolitan University, 7-2-10 Higashiogu, Arakawa-ku, Tokyo, 116-8551, Japan.


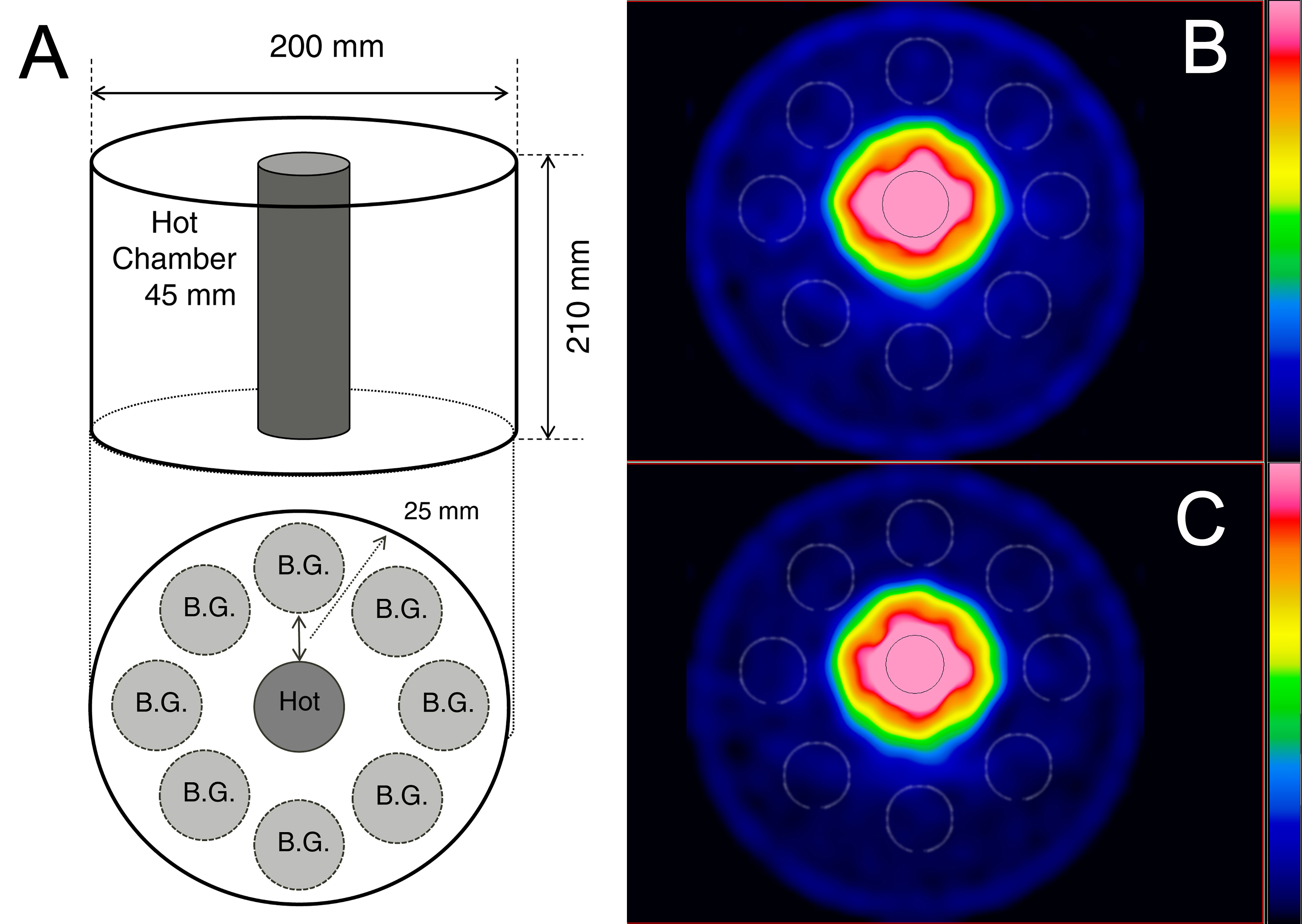


Additional file 1: Figure S1 Configuration of a cylindrical phantom (A) and summed transaxial images (slice thickness, 14 cm) at 84 keV with MEGP (B) and HEGP (C) collimators. The phantom was filled with water, and a hot rod (2.0 kBq/mL) was embedded along the phantom axis. Circular regions-of-interest were placed on the hot rod and background.

MEGP, medium-energy general purpose; HEGP, high-energy general purpose


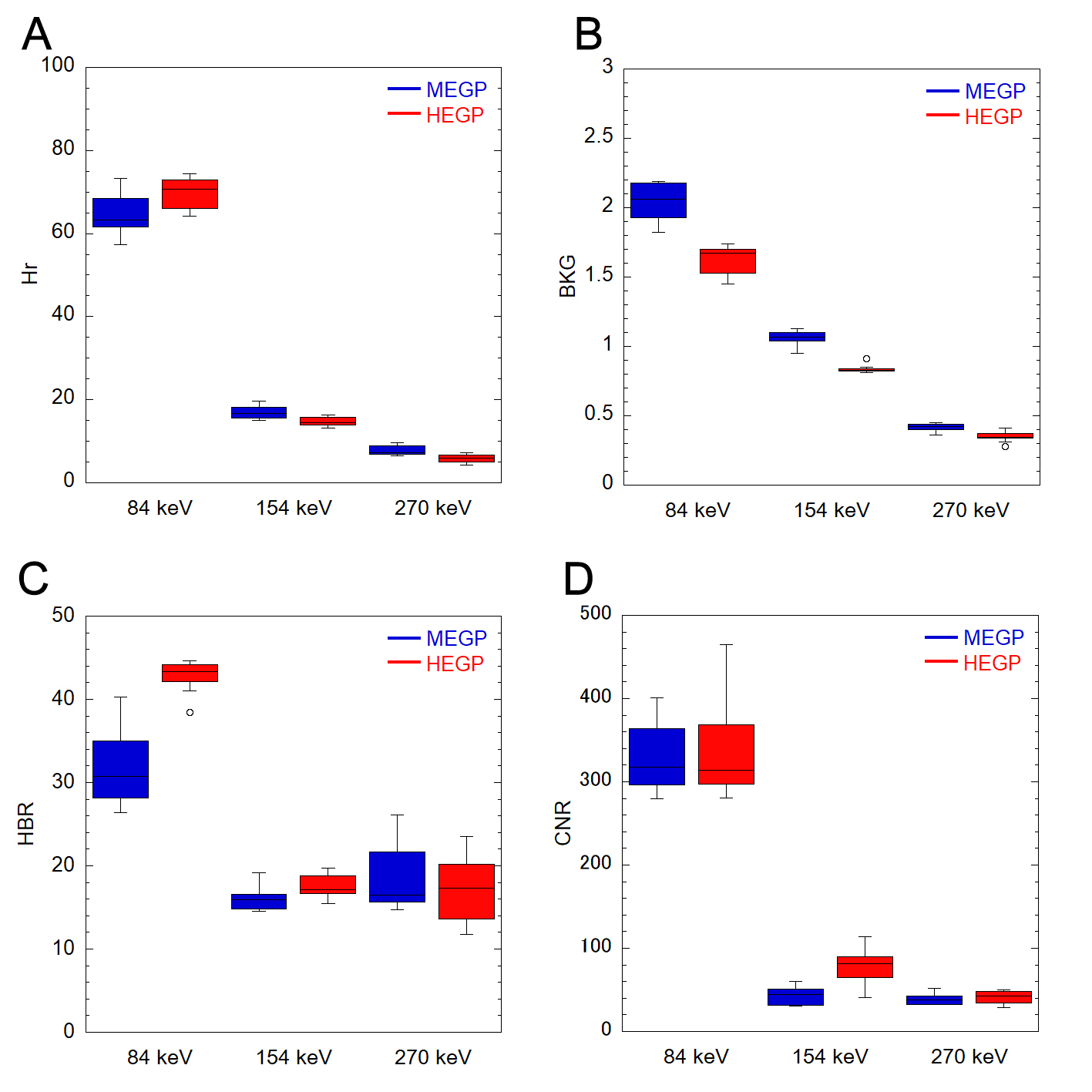


Additional file 1: Figure S2**.** SPECT counts in a hot rod (Hr) and background (BKG), hot rod-to-background ratio (HBR) and contrast-to-noise ratio (CNR) at 84 keV ± 20%, 154 keV ± 10% and 269 keV ± 5%.
